# Exposure index in digital radiography and its dependence on acquisition parameters, anatomy, and manufacturer

**DOI:** 10.1002/acm2.70331

**Published:** 2026-01-14

**Authors:** Ioannis A. Tsalafoutas, Shady AlKhazzam, Mohammed Hassan Kharita

**Affiliations:** ^1^ Medical Physics Section OHS Department Hamad Medical Corporation Doha Qatar; ^2^ Present address: Medical Physics Department General Anticancer Oncology Hospital of Athens ‘Agios Savvas’ Athens Greece

**Keywords:** Anthropomorphic phantoms, deviation index, digital radiography, exposure index, image quality, target exposure index

## Abstract

**PURPOSE:**

The exposure index (EI), the target exposure index (EI_T_), and the deviation index (DI) have been defined in the IEC Standard 62494‐1 Ed.1 2008‐08. This study investigates the impact of certain acquisition parameters, the imaged anatomy, and the manufacturer's specificities on the EI of radiological images and how these may affect EI_T_ setting procedure.

**METHODS:**

Images were acquired using two digital radiography (DR) systems of two different manufacturers, using aluminum attenuators and an anthropomorphic phantom. Acquisition parameters like the tube potential (kVp), the tube loading (mAs), the exposure time, the automatic exposure control (AEC) system settings (sensor and dose level selection), the grid (with or without), the additional filtration, the field size, and the imaged anatomy were varied and their effect on the EI was quantified separately for each system.

**RESULTS:**

EI is linearly related to the incident air kerma (IAK) on the detector as expected (by definition). For constant IAK, EI increases with increasing kVp. While EI in general is reduced in the presence of scatter, this may not always be the case. Under AEC operation, even the exposure time can make a difference. EI is strongly affected by the imaged anatomy in combination with the AEC sensor and field size selections, the examination protocol, and the manufacturer.

**CONCLUSIONS:**

Many parameters affect the EI calculation apart from IAK. Among them, the most important are the imaged anatomy and the manufacturer. Since the EI calculation is a complex procedure, setting of the EI_T_ values should be done with caution on a per‐examination and manufacturer basis, since the values that apply for one digital system are not always applicable to another. Furthermore, when EI is used as an image quality tool, a DI variation of at least ±2 should be allowed before a possibly meaningful red flag is activated.

## 1 INTRODUCTION

It is well known that unlike the classic screen‐film radiography systems where the film darkness was related to dose, in digital radiography this is no longer the case. The digital image receptors (detectors) can be irradiated to a much larger incident air kerma (IAK) than their predecessors and still look perfectly fine in terms of image darkness, that is, with no sign of overexposure. However, the unlinking of image darkening from IAK posed the danger of systematic use of increased IAK to reduce noise and improve image quality.^1^, ^2^, ^3^ In answer to this problem, the digital x‐ray system manufacturers devised various exposure indicators to inform the users about the IAK on the detector, which, however, differed much from each other and were not always easy to use in clinical practice.^4^

For this reason, a universal exposure index (EI) was defined in the IEC Standard 62494‐1 Ed. 1 2008‐08.^5^ This EI is 100 times the IAK value in µGy on the image receptor when the RQA‐5 x‐ray beam quality is used. Within the same IEC standard, the deviation index (DI) is also defined as follows: DI = 10 × log_10_[EI/EI_T_], where EI_T_ is the target EI. An image with EI equal to (or similar) EI_T_ is assumed to have an adequate statistical quality (low enough quantum noise level) so that it is diagnostic. The advantage of DI is that the user does not have to know by heart the value of EI_T_ to determine whether the IAK on the detector and thus image quality is adequate. If the DI value is within certain limits, e.g., within ±2 or within ±3, it can be considered good or acceptable, respectively.

Previous attempts that have been made to define which are the EI_T_ values that should be set for various examinations ended up suggesting a broad range of EI_T_ for certain examinations and manufacturers, which, however, is far too general and far too broad to be practically useful.^6^, ^7^ Presumably this could be attributed to the various conditions with which the same examinations are performed in various facilities (like different kVp and automatic exposure control (AEC) settings) and to a variety of problems that may lead to a calculation of an incorrect EI value. The study by Don et al.^8^ provides a concise description of the various problems involved in the calculation of EI in clinical images and the subsequent problems in setting proper EI_T_ values.

In this study, the dependence of EI on certain acquisition parameters is investigated using various attenuators, phantoms and acquisition parameters, and two digital x‐ray systems from different manufacturers.

## 2 MATERIAL AND METHODS

**FIGURE 1:**
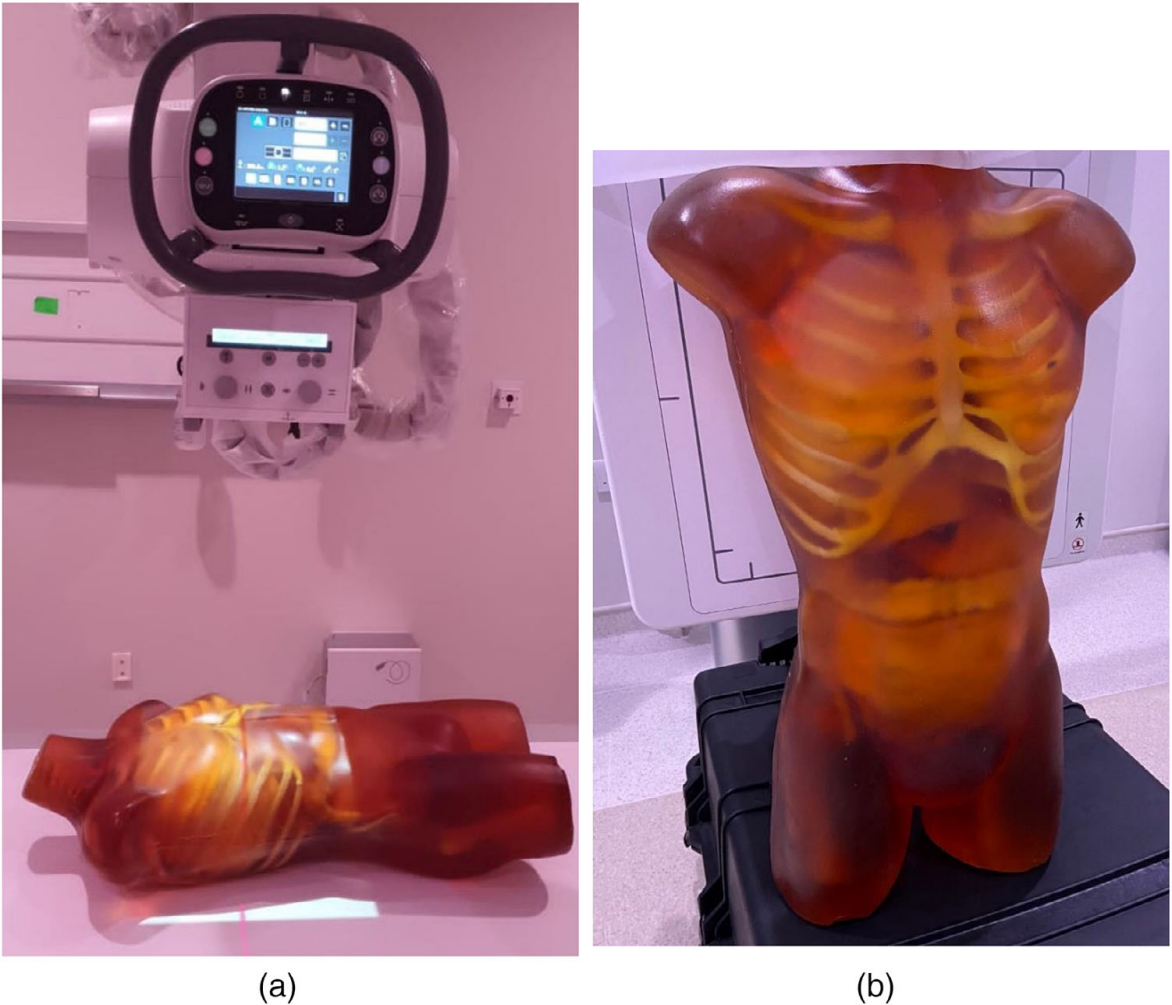
Positioning of the anthropomorphic phantom: (a) in the horizontal table (TB), (b) in front of the chest bucky (CB).

Two experimental sessions were conducted, one using flat fields and aluminum (Al) attenuators of 20‐ or 21‐mm thickness, and one using an anthropomorphic phantom (Adult Torso for x‐ray, CT, MRI, True Phantom Solutions) shown in Figure 1a,b. The first session represents the quality control (QC) conditions, where the detector and AEC system calibration are tested, and the second the clinical practice conditions.

All measurements made with both the Al attenuators and the anthropomorphic phantom were carried out in a single GE system (Discovery XR656HD, GE HealthCare, Chicago, Illinois, USA) and a single Philips Digital Diagnost system (Philips Medical Systems B.V., Eindhoven, Netherlands), henceforth referred to as GE and Philips, respectively. However, the measurements with the Al attenuators were repeated in another four additional identical GE radiograph systems and another three identical Philips radiography systems. For all IAK measurements the radiography dosimetric sensor of the RaySafe X2 multimeter (Fluke Corporation, USA) was used, henceforth referred to as dosimeter. It should be noted that for all radiography systems used in this study, the calibrations of the table (TB) and chest bucky (CB) detectors have been tested using the IEC standard methodology in the context of the routine QC procedure and were found to be within limits (differences were within ±20%).

### 2.1 Measurements with flat fields and 20/21 mm aluminum attenuator

In the first experimental session (QC conditions’ session), measurements of EI using various acquisition settings were performed using the Al attenuator that is provided by the manufacturer for the detector and AEC calibration (20 mm Al for GE systems and 21 mm for Philips systems). Four sets of exposures were performed for both the TB and CB of each radiography system. For all exposures performed at the TB, the Abdomen AP examination protocol was used, while for the exposures performed at the CB, the Chest AP examination protocol was used.

The source‐to‐image receptor distances (SID) used in clinical practice for each system were employed (SID for TB was 100 cm for GE and 115 cm for Philips, and for CB 180 cm for both GE and Philips). Tube potential (kVp) values of 70, 100, and 120 kV were employed, and when the exact kVp values were not available, the closest setting was used (for GE systems, 102 and 125 kV were used).

In the first set of measurements, the detector was retracted, the dosimeter was inserted in the center of the detector holder, and the IAK values for all three kVp selections were measured at the detector position (IAK_detector_) using the same manual mAs settings (20 mAs), and the IAK_detector_ values per mAs were calculated [IAK_detector/mAs_(kVp)]. In this way, for all subsequent exposures (sets 2–4) performed with the detector in place (the dosimeter was removed), the IAK at the detector level could be calculated by the following equation:

(1) IAKdetector(kVp,mAs)=mAs×IAKdetector/mAs(kVp)

However, for the Philips systems where the detectors could not be removed, IAK measurements at the detector level were not feasible. In this case, IAK measurements were made with the dosimeter positioned on the table, and the IAK_table_ values per mAs were calculated [IAK_table/mAs_(kVp)]. To derive the respective values at the detector level, corrections had to be applied for the attenuation of the x‐ray beam by the table, the grid, and the difference between SID and source‐to‐dosimeter distance (SDD) using one of the following equations:

(2a) $$\begin{array}{cc} & \mathrm{IAKdetector}(\mathrm{kVp},\mathrm{mAs})=\mathrm{mAs}\;\times \;\mathrm{IAKtable}/\mathrm{mAs}(\mathrm{kVp})\\ & \;\times \mathrm{TFtable}\times \mathrm{TFgrid}\times (\mathrm{SDD}/\mathrm{SID})2 \end{array}$$

(2b) $$\begin{array}{cc} & \mathrm{IAKdetector}(\mathrm{kVp},\mathrm{mAs})=\mathrm{mAs}\;\times \;\mathrm{IAKtable}/\mathrm{mAs}(\mathrm{kVp})\\ & \;\times 1/\mathrm{BF} \end{array}$$

In the above equations, TF_table_ stands for the transmission factor of the table, TF_grid_ stands for the transmission factor of the grid, and BF stands for the bucky factor. Though for the Philips systems, bucky factor values are provided by the manufacturer for all different grids available (one value per grid for all kVp values), it was preferred to calculate the transmission factors of the grid and the table for each different kVp value from actual measurements. The TF_grid_ for each kVp value was obtained by performing exposures using AEC and the central AEC sensor activated, with the dosimeter positioned at the table, at a position well under the central AEC sensor, so that it did not interfere with the AEC operation. The TF_grid_ was calculated as the average value of the displayed mAs values selected by the AEC and the measured IAK ratios (value without grid/value with grid).

The TF_table_ is routinely obtained from IAK measurements with exposures performed using manual mAs, the grid and detector retracted, and the dosimeter positioned first on the table and then at the detector position. It is calculated by dividing the IAK value with the dosimeter on the detector position by the IAK value with the dosimeter on the table and multiplying by (SID/SDD)^2^ to correct for the inverse square law. Since the table transmission of the Philips systems could not be calculated (because the detector could not be removed), the TF_table_ values determined in one of the GE radiography systems were used as a first approximation.

In the second set of measurements, flat field images were obtained for all three kVp selections by performing exposures made with manual mAs (5 mAs), and the EI values displayed (EI_displayed_) were compared with the EI values calculated (EI_displayed_) using the results from the first group measurements using the following equation:

(3) EIcalculated=100×IAKdetector(kVp,mAs)

The percentage differences between calculated and displayed EI values (ΔΕΙ) were determined using the following equation:

(4) ΔEI=100×(EIcalculated−EIdisplayed)/EIdisplayed

In the third set of measurements, flat field images were obtained with all three kVp selections, performing exposures under AEC (central sensor activated), at the preset target IAK (IAK_T_) setting of the AEC, and the EI values displayed were compared with those calculated using Equations (3) and (4). Note that in all GE radiography systems the preset IAK_T_ was about 2.5 µGy (i.e. 400 relative speed). However, in Philips systems, it was known (from routine QC tests) that IAK_T_ values had been found to be closer to 2.0 µGy, but no adjustments were carried out since no complaints about the image quality of clinical images had been reported. Finally, in the fourth set of exposures, the mAs and EI values of images acquired using each different AEC sensor separately (with 70 kV only) and all their possible combinations were recorded to investigate for any possible AEC sensor relative response imbalances.

### 2.2 Measurements with the anthropomorphic phantom

In the second experimental session (clinical conditions session), the anthropomorphic phantom was used. The GE system has numerous preset examination protocols for a variety of radiographic projections. Each examination protocol has various default settings regarding the kVp value, the focus size, the respective image receptor (TB or CB), the AEC sensor combination activated, the use of grid or not, etc. Each examination protocol also includes an embedded postprocessing algorithm that is designed to optimize the image quality of clinical images. As can be seen in Tables 1a and 1b where the preset protocols for the Chest AP and Ribs AP and the main relevant acquisition parameters are tabulated, the default values of the protocol parameters change depending on the patient size (e.g., kVp, grid yes/no, AEC sensor combination), and they are offered three size selections (small, medium, and large) for pediatric (p) and adult patients (a), which henceforth will be referred to as pS, pM, pL, aS, aM, and aL. The only advanced characteristic that is not included in the purchased GE system configuration is the automatic setting of the radiation field size to the preset dimensions of each examination protocol (the field size must be manually adjusted by the operator). The Philips system has similar characteristics to the GE system plus the preset field size adjustment. The only thing that changes is patient size categorization (newborn, small, normal, large, and extra‐large). However, unlike GE, which was delivered with preset values of EI_T_ for all exams, Philips has no preset values of EI_T_ yet, so the calculation of DI is not feasible.

**TABLE 1a:** Preset settings in the GE system for the Chest AP protocol for the TB and different patient size selections, and resulting entrance dose, KAP, EI, target EI, and DI values when imaging the chest of the anthropomorphic phantom.

Patient size	pS	pM	pL	aS	aM	aL
Focus size	Small	Small	Small	Small	Large	Large
Grid	*No*	*No*	No	Yes	Yes	Yes
kVp	60	70	80	90	100	110
mA	80	80	80	200	320	500
AEC sensors	C	LC	LR	LR	LR	LR
KAP (dGycm^2^)	2.75	1.44	1.00	2.51	2.32	2.24
EI	224.2	178.6	162.9	176.3	195.4	201.2
Target EI (EI_T_)	100.8	102.9	105	109.7	114.3	116.7
DI	3.5	2.4	1.9	2.1	2.3	2.4

*Note*: The SID was 100 cm, the field size was kept constant at 423 mm × 426 mm, speed was 400, and added filtration was zero.

Abbreviations: AEC, automatic exposure control; DI, deviation index; EI, exposure index; SID, source‐to‐image receptor distances; target EI, target exposure index.

**TABLE 1b:** Preset settings in the GE system for the Ribs AP protocol (TB) and different patient size selections, and resulting entrance dose, KAP, EI, target EI, and DI values when imaging the chest of the anthropomorphic phantom.

Focus size	Small	Large	Large
Patient size	aS	aM	aL
Grid	Yes	Yes	Yes
kVp	70	75	80
mA	320	400	500
AEC sensors	C	C	C
KAP (dGycm^2^)	19.81	15.34	12.65
EI	751	723	713
Target EI (EI_T_)	294	297	300
DI	4.1	3.9	3.8

*Note*: The SID was 100 cm, the field size was kept constant at 423 mm × 426 mm, speed was 400, and added filtration was zero.

Abbreviations: AEC, automatic exposure control; DI, deviation index; EI, exposure index; SID, source‐to‐image receptor distances; target EI, target exposure index.

#### 2.2.1 AEC sensor combination selection

AEC systems commonly use three sensors (left (L), central or middle (C), and right (R)) positioned under the radiographic table and the chest stand that can be activated one at a time, all together, or any two of them. Depending on the x‐rayed anatomy, the sensors may be below patient anatomic structures with different thickness and/or density, thus requiring different mAs to reach the IAK_T_. Therefore, the activated AEC sensor combination may affect the mAs selection and subsequently affect also the EI and patient dose (expressed using surrogate quantities like Kerma‐Area Product (KAP), or entrance dose). To document the differences that could occur in EI because of using different AEC combinations in different examinations, four different acquisition setups were used for the GE system used for imaging of the anthropomorphic phantom (later referred to with the code GE‐4). These correspond to four different examinations, all with SID = 100 cm; in the first three, the anthropomorphic phantom was positioned on the TB (see Figure 1a), while in the fourth it was positioned standing in front of the CB (see Figure 1b). The phantom anatomies imaged were the chest (TB) and the abdomen and pelvis (TB, CB). For the Philips system for imaging of the anthropomorphic phantom (later referred to with the code PH‐4), two different acquisition setups were used (SID = 110 cm), which correspond to two different examinations performed with the TB, Chest AP and Abdomen/Pelvis AP. For each one of these six different examination setups, images with all different AEC sensor combinations were acquired.

#### 2.2.2 Anatomy imaged with different radiation field size protocol selections

Images of the chest part and the abdomen parts of the anthropomorphic phantom were acquired using different protocols and different radiation field sizes and different AEC settings. In the Philips system, different additional filtrations were also used.

#### 2.2.3 Short exposure time‐related issues

To investigate the effect of exposure time on EI, the chest part of the anthropomorphic phantom was positioned on the TB and the Chest AP examination preset protocol was used. For the GE system, the mA setting varied from 10 to 400 mA (10, 25, 50, 100, 200, and 400 mA). For the Philips system, the mA value setting varied from 10 to 620 mA (10, 20, 80, 150, 300, 500, and 620 mA), and the resulting mAs, EI, and KAP values were all recorded. It must be clarified that all images were acquired under AEC, but the mA was restricted (manually selected) so that the AEC system selected only the exposure time.

#### 2.2.4 Effects of examination protocol and patient size selection

Modern x‐ray systems offer a default examination protocol that is supposed to be applicable for standard‐sized adults only. For patients of different sizes and/or ages, modified versions of this protocol are often offered that most commonly vary in terms of kVp (larger for large patients), but may also vary regarding mA, focus size, AEC sensor combination, and the use or non‐use of grid, since grid is not used for pediatric patients nor in general for thin anatomic structures like hands, arms, etc. Since the anthropomorphic phantom presents a medium‐sized adult patient, it was thought that it would be interesting to investigate how the selection of an examination protocol designed for a different patient size could affect the EI and patient dose‐related metrics in the case of an average adult patient.

For GE, this investigation was carried out using the Chest AP (pediatric and adult patients) and Ribs AP (adult patients only) examination protocols to image the chest part of the anthropomorphic phantom positioned on the TB. For the Philips system, only the Chest AP examination protocol was investigated. The preset Chest AP and Ribs AP examination protocol settings of the GE and the Philips systems for different patient sizes were used to image the chest region of the anthropomorphic phantoms. The protocol settings were recorded and their effect on EI values and on dose metrics was studied.

## 3 RESULTS

### 3.1 Results from flat field images acquired with the 20/21 mm Al attenuator

**FIGURE 2:**
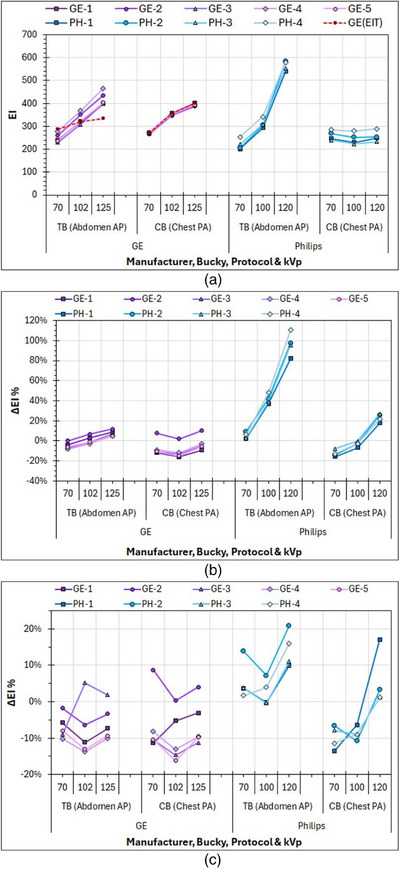
Results of measurements with flat fields in TB and CB using the Al phantoms and different kVp in five GE and four Philips systems: (a) Displayed EI values of images acquired under AEC, (b) differences between calculated and displayed EI of images acquired under AEC, and (c) differences between calculated and displayed EI in images acquired with constant mAs. AEC, automatic exposure control; EI, exposure index.

In Figure 2a, a distinct difference between GE and Philips can be observed regarding the EI values of the flat field images acquired under AEC. The GE system presented a distinct increase of EI values with kVp, whereas Philips presented this increase only for TB, as for the CB, the EI values did not vary much with kVp. It must be emphasized that the EI data points for 70 kV in Figure 2a were acquired using an attenuator of 20/21 mm Al (RQA‐5 x‐ray beam conditions but with the grid in place and the interference of the TB and CB tabletop) and therefore exhibit the differences between the five GE systems (10 detectors) and four Philips systems (8 detectors) that have arisen as a result of combined variations in detectors’ EI calibration and AEC systems target IAK settings. Indeed, for GE systems, the displayed EI values ranged from 230 to 279 (21%) for TB and from 266 to 272 (2%) for CB, while for Philips systems, EI ranged from 201 to 253 (26%) for TB and from 240 to 285 (19%) for CB.

In Figure 2b, it is evident that for these images, differences between displayed and calculated EI values (ΔEI%) for both TB and CB were small for the GE systems (ranging from −16% to 12%), slightly larger for the Philips systems (ranging from −16% to +27%) regarding the images acquired in CB, but for the images acquired in TB, ΔEI% gradually increased from 2%–9% at 70 kV to about 37%–48% at 100 kV and reached 82%–110% at 120 kV. However, in Figure 2c, it is apparent that for the flat field images acquired using manual mAs, the respective ΔEI% values were much smaller for all kVp values and for both TB and CB. For GE systems, ΔEI% values ranged from −16% to 9%, and for Philips systems, they ranged from −11% to +21%.

Regarding GE systems, the increase of EI values with kVp was expected as can be seen from the preset EI_T_ values for the Abdomen AP (TB) and Chest AP (CB) examinations, also shown in Figure 2a with red dashed lines [GE(EI_T_)], since the detector response for the same IAK increases with kVp. However, while the increase of EI values with kVp for the flat field images acquired in CB was closely following the respective pattern of EI_T_ values, for TB, the increase was much steeper than that expected.

Trying to explain the large ΔEI% values observed in Philips systems with increasing kVp values, only for the flat field images acquired in TB, it was finally understood that this was attributed to a combination of limitations in the AEC operation and x‐ray generator occurring when the selected exposure times are well below 10 ms. To report some numbers, under AEC operation, the exposure times for GE systems where the mA was set to 100 mA (the AEC selects only the exposure time) were about 20, 6, and 3–4 ms for images acquired in TB and 70, 20, and 12 ms for images acquired in CB. The respective exposure times for the Philips systems where the mA was set to 100 mA were about 20, 5, and 3 ms for images acquired in TB (PH‐3, PH‐4) and 100, 15, and 9 ms for images acquired in CB (PH‐1, PH‐2, PH‐3, and PH‐4). For the Philips systems where the mA was set at 400 mA for images acquired in TB (PH‐1, PH‐2), the respective exposure times were about 6, 2, and 1.5 ms.

By positioning a dosimeter on the TB of the Philips systems (at a position not interfering with the central AEC sensor) and measuring the IAK/mAs ratios under AEC and using manual values (the closest manual mAs to the AEC‐selected value was used with the smallest exposure time allowed, which was 10 ms), it was seen that for exposures under AEC where exposure times were smaller than 10 ms, the IAK/mAs ratio gradually reduced compared to the respective ratio obtained with manual mAs (for all kVp values), dropping down to about 50% for exposure times of about 2 ms. This indicates that the generator output is significantly reduced when the exposure times become too small and comparable with the generator rise time (∼1 ms) during which the kVp is gradually increased to reach the selected kVp value. Note that during this kVp increase to the set value, softer x‐rays are emitted, which are more strongly absorbed by the attenuator. Since the calculated EI values were determined using the IAK_detector_ (kVp,mAs) values derived using manual mAs (i.e., for normal x‐ray output), it is understood that they can be overestimated by up to about 100%, and this explains the large ΔEI% values observed in Figure 2b. However, it does not explain the increase in the actual (displayed) EI values with kVp increase observed for the images acquired in TB for all Philips systems and GE systems. The increase was much larger for Philips systems compared to GE systems, but even in GE systems, this increase was still larger than anticipated. This indicated that there should be a second parameter implicated.

This parameter is the AEC minimum response time, which is of the order of 1 ms and which introduces a delay in the switch‐off of the generator. This represents the shortest time in which the AEC system can terminate the exposure once it detects that the desired IAK_T_ has been reached. To explain the effect of this delay with an example, if an exposure under AEC is performed using a kVp value of 120 kV without an attenuator and only 0.2 ms are required for the IAK to reach the IAK_T_ of 2.5 µGy (switch‐off dose), then the exposure duration will still be at least equal to the AEC response time, and assuming that this is 1 ms, then the IAK reaching the detector will be about five times more and so will be the EI value (ignoring the aforementioned problems of generator rise time).

To explore these effects, additional measurements were performed in both the GE and Philips systems. Flat field images were acquired using the Abdomen AP protocol, with the grid retracted, without attenuator, using different kVp, SID (70, 100, and 120 cm) and mA selections, with a dosimeter positioned on the table surface. Images with exposure times as low as 0.1 ms for the GE system and 0.2 ms for the Philips system were derived (using the smallest SID, the higher kVp, and the higher mA allowed), suggesting that the AEC response time is very low. Even so, the maximum IAK values recorded were 28 µGy for the GE system and 56 µGy for the Philips system, and the respective EI values were 830 and 2660. The combined effect of the generator rise time and AEC response time can be understood from the measurements made using 81 kV and 100 cm SID and two different mA values (50 and 400 mA). The corresponding exposure times, IAK on the table and EI for the low mA selection were 2.46 ms, 7.27 µGy, and 376, and for the large mA selection, 0.57 ms, 11.9 µGy, and 513. For the Philips system, the respective numbers for the low mA selection were 2.8 ms, 9.75 µGy, and 359, and for the large mA selection, 0.7 ms, 17.9 µGy, and 702. From the kVp and output waveforms, it was verified that the generator rise time was about 1.2 ms for the GE and 0.8 ms for the Philips unit.

Finally, regarding relative sensor response, in GE systems differences between EI and mAs values did not exceed 6%, while in Philips systems differences of up to 20% were observed, with the left and right sensors resulting in general in larger mAs and EI values than the central one.

### 3.2 Results from anthropomorphic phantom images

#### 3.2.1 AEC sensor combination selection

**FIGURE 3:**
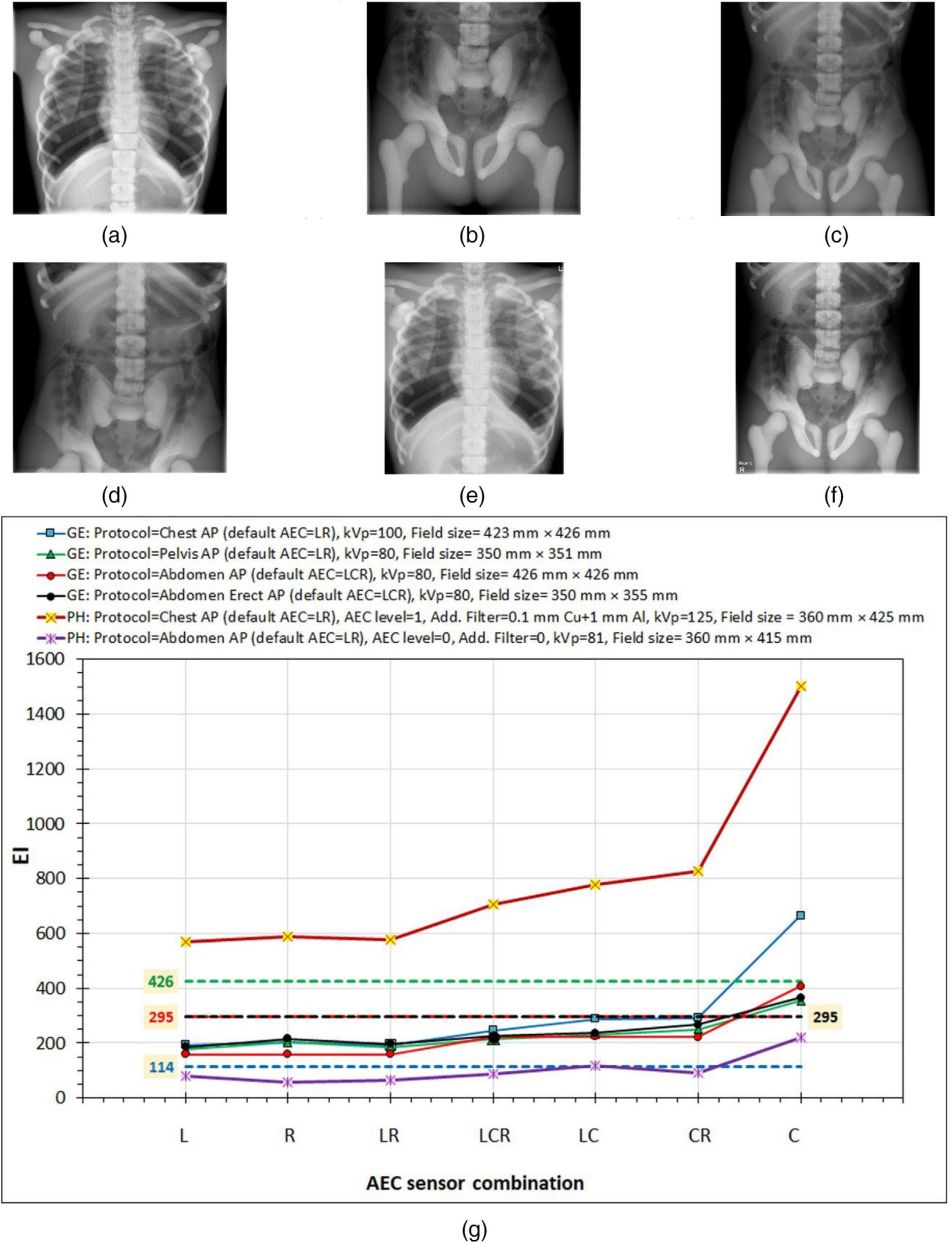
The effect of the sensor combination settings (L, R, and C stand for the left, right, and central AEC sensors being activated) on EI values. (a) GE (TB): Protocol = Chest AP (default AEC = LR), kVp = 100, field size = 423 mm × 426 mm. (b) GE (TB): Protocol = Pelvis AP (default AEC  =  LR), kVp = 80, field size = 350 mm × 351 mm. (c) GE (TB): Protocol = Abdomen AP (default AEC = LCR), kVp = 80, field size = 426 mm × 426 mm. (d) GE (CB): Protocol = Abdomen erect AP (default AEC = LCR), kVp = 80, field size = 350 mm × 355 mm. (e) PH (TB): Protocol = Chest AP (default AEC = LR), kVp = 125, field size =  360 mm × 425 mm. (f) PH (TB): Protocol = Abdomen AP (default AEC = LCR), kVp = 81, field size =  360 mm × 415 mm. (g) The variation of EI with AEC sensor combination activated for different examinations in the GE and Philips systems. Dashed lines represent the EI_T_ values for the respective examinations in the GE system. AEC, automatic exposure control; EI, exposure index; EI_T_, target exposure index.

The EI variations with AEC sensor combination selection for the six examination setups studied, which are shown in Figure 3e,f, are shown in Figure 3g. To interpret the EI variations, one must understand where the position of each AEC sensor is in each one of the six images. Using as an example the Chest AP image (Figure 3a) acquired in the GE system, the central sensor (C) is under the spinal cord (at the image center), the right AEC sensor is under the right lung (slightly above image mid‐height and ∼10 cm from the vertical midline), and the left sensor is under the left lung and the heart. As can be seen in Figure 3g, in the curve corresponding to Figure 3a, the right sensor resulted in slightly larger EI value than the left sensor, which could be attributed to differences in the attenuation caused by the overlying anatomy leading to slightly larger mAs, but it could also be due to small differences between left and right sensors’ responses. When the central AEC sensor was activated along with both the left and right sensors or only one of them, the EI increased because mAs increased. This is because the contribution of the central sensor to the sum of the IAK_T_ values is smaller than the other two sensors (the sum of the IAK values in every sensor divided by the number of activated sensors should be about 2.5 µGy), so the mAs had to be increased to compensate for the reduced IAK at the central sensor. In all four examinations in the GE system, the maximum EI (and mAs value) was observed when only the central sensor was activated. However, this is more prominent in the chest x‐ray, where the biggest attenuation difference exists between the anatomical structures that lie above the central and the lateral AEC sensors.

For the Philips system, the variation patterns for the Chest AP and Pelvis AP examinations with AEC sensor selection are similar to the respective patterns observed for GE. However, for Philips there was a large difference between the EI values between the different anatomies imaged, since the EI values for Chest AP were 6.6–10.2 times larger than the respective values for Pelvis AP. While this could be attributed to the different anatomies imaged, one additional exposure of the same abdomen and pelvis anatomy shown in Figure 3f, but selecting as examination protocol the Chest AP instead of the Abdomen AP, resulted in a much larger EI value, very similar to that obtained when imaging the chest anatomy (Figure 3e), suggesting that for the Philips system, the post‐processing algorithm can greatly affect the EI values.

What is also interesting to mention regarding Figure 3g is that while the EI values obtained with this phantom for the four different examinations in the GE system are quite similar to each other (with the only exception being the effect of the central AEC sensor), they were much different than the default EI_T_ values for the Chest AP, the Abdomen AP/Abdomen Erect AP, and the Pelvis AP (shown with colored dashed lines).

#### 3.2.2 Anatomy imaged with different radiation field size and protocol selections

**FIGURE 4:**
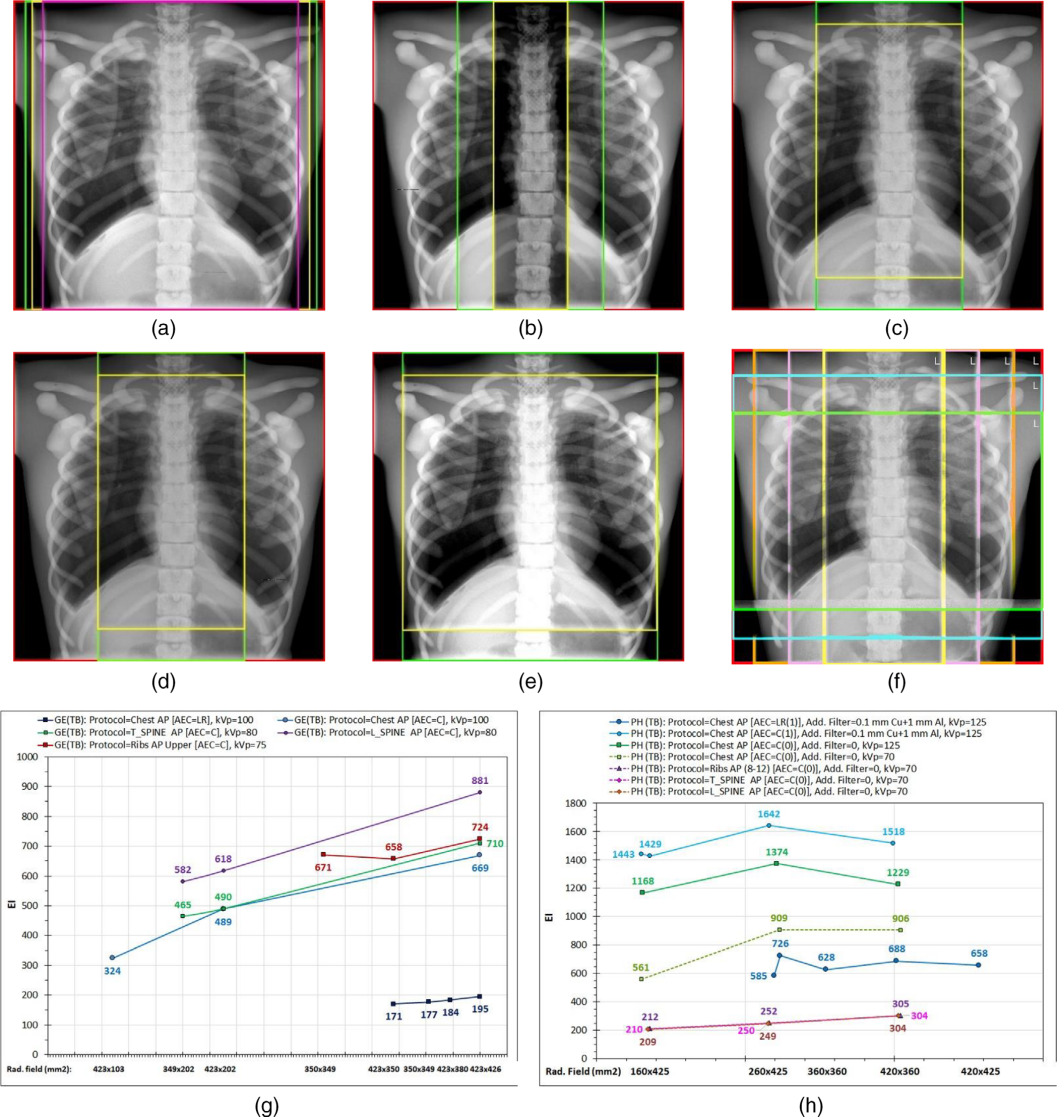
The effect of protocol and field size selection on EI values using the chest part of the phantom. The different color frames indicate the different field sizes used. (a) GE (TB): Protocol = Chest AP (AEC = LR), kVp = 100, speed = 400. (b) GE (TB): Protocol = Chest AP (AEC = C), kVp = 100, speed = 400. (c) GE (TB): Protocol = T_SPINE AP (AEC = C), kVp = 80, speed = 400. (d) GE (TB): Protocol = L_SPINE AP (AEC = C), kVp = 80, speed = 320. (e) GE (TB): Protocol = Ribs AP upper (AEC = C), kVp = 75, speed = 400. (f) PH (TB): Protocol = Chest AP and Ribs AP (8–12), various additional filter selections, kVp and AEC selections, speed = 400. AEC, automatic exposure control.

Regarding GE, Figure 4g shows that for the Chest AP protocol where the default AEC sensor combination is activated (LR), the more the left and right field edges were trimmed (Figure 4a), the more the EI decreased (the mAs remained roughly the same), but the differences were small (see blue data points in Figure 4g). The reduction of EI can be explained by considering that reducing the field width excludes more highly exposed pixels than low‐exposed pixels (considering always only the pixels considered as clinical content and not pixels corresponding to bare x‐ray beam).

For the Chest AP protocol again but with the central AEC sensor activated only (Figure 4b), reducing the field width from 43 to 20 cm, as in the thoracic spine examination (T_Spine AP), and then down to 10 cm decreased the EI values by 30% and 52%, respectively. This is justified by the reduction in the ratio of pixels with larger exposure to pixels with lower exposure because of reduction of the percentage of parenchymatic tissue imaged. Comparing the EI values of Figure 4g between the images with the largest field sizes of Figure 4a,b, the EI value is 3.4 times larger with the C than the LR sensors activated (because of a respective 3.1 times increase in mAs) since above that central AEC sensor lies the highly attenuating spinal cord. For the actual T_Spine AP examination protocol (Figure 4c), the EI values are similar to those of the previous examination (Figure 4b). Any differences in EI values could be attributed to the change of kVp (80 kV instead of 100 kV before), which changes the relative exposure differences of the pixels that image the parenchyma and the spine.

Using the Lumbar Spine (L_spine) AP examination protocol (Figure 4d), which uses the same kVp and AEC sensor with the T_spine AP protocol, resulted in increased mAs and EI values, but only because the speed of this protocol is 320 and thus both the mAs and EI were increased by the 400/320 ratio. Finally, the use of Ribs AP protocol (Figure 4e) resulted in slightly increased EI values with respect to the previous protocols, and it was only slightly reduced with small width and height field size trimmings. These results suggest that for the GE system, the post‐processing algorithm embedded in each examination protocol is not expected to affect the EI calculation, which is made using the raw (for‐processing) images.

Regarding the Philips system, the chest anatomy shown in Figure 4f was imaged using various radiation field sizes, AEC sensor and dose level selections, different additional filters, different kVp, and different examination protocols. As can be seen in Figure 4h, the field size did always affect the EI values in all cases. What is more interesting, though, is that when focusing on the four last curves where the same acquisition conditions were employed (kVp = 70, added filter = 0, AEC = C(0)), it is obvious that for all field sizes the Chest AP protocol always resulted in much larger EI values compared to the other three protocols (Ribs AP (8–12), T_Spine AP, and L_Spine AP), which produced roughly the same EI values.

**FIGURE 5:**
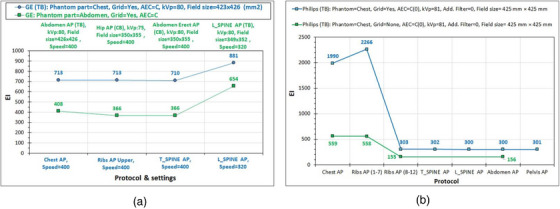
The effect of protocol selection on EI values when imaging the same anatomy. (a) GE (TB), anatomy: chest (in blue) and abdomen (in green). (b) Philips (TB), anatomy: Chest AP. EI, exposure index.

To isolate the effect of examination protocol selection from the field size selection, the EI values of images of the same anatomy (chest and abdomen parts of the anthropomorphic phantom) that were obtained with similar conditions but with different examination protocols were compared and the results are presented in Figure 5a. In this figure, it is clearly shown that for the GE system, if all the other conditions are kept constant, the postprocessing algorithm embedded in each examination protocol does not affect EI calculation. Thus, examination protocol selection affects EI only indirectly because parameters like AEC sensor selection and speed may differ. On the contrary, for the Philips system, it was found that protocol selection may strongly affect EI values, as shown in Figure 5b. In fact, from the protocols tested, two distinct groups were identified: the first contains the Chest AP and the Ribs AP (1–7) protocols, which result in the larger EI values (1990 and 2260, respectively), and the second contains all the rest of the protocols tested, for which the EI values appear to be six to seven times smaller but more closely related to the IAK_T_ than that of the first group. The lower EI values obtained in the Philips system when the grid was retracted should also be noted.

An interesting detail related to Figure 5b that should be mentioned is that in repeated acquisitions of the chest anatomy with the Chest AP using 81 kV, under AEC with the central AEC sensor activated and with the grid in place, presented identical mAs values (10 mAs), whereas the acquisitions with Ribs AP (1–7), Ribs AP (8–12), and Abdomen protocols all gave 11.3 mAs. On the contrary, without the grid, all protocols gave the same mAs value (1.78 mAs). It was finally understood that the reason for the reduced mAs value of the Chest AP examination protocol was the backup limit against overexposure (10 mAs), since in this system Chest AP is supposed to be performed with 125 kV and the left and right AEC sensors activated, so values above 10 mAs are abnormal. So, if the Chest AP image EI is corrected using the mAs ratio (11.3/10), it is deduced that without the backup limit, the EI value would be 2249, which is almost equal to the EI of Ribs (1–7) AP image.

#### 3.2.3 Short exposure time‐related issues

Regarding the effect of exposure time on EI, it has been proven to be negligible for the GE system. Though when imaging the chest anatomy in the TB using the Chest AP protocol exposure and different mA values, the exposure times ranged from 3.4 to 123 ms, and the EI values were within the 192–196 range. However, for smaller exposure times, the KAP gradually increased to up to 14% for the smallest exposure time (largest mA value) compared to the largest exposure time (smallest mA), something that could be attributed to the reduced kVp and the accordingly reduced x‐ray beam output during the generator rise time mentioned earlier.

**FIGURE 6:**
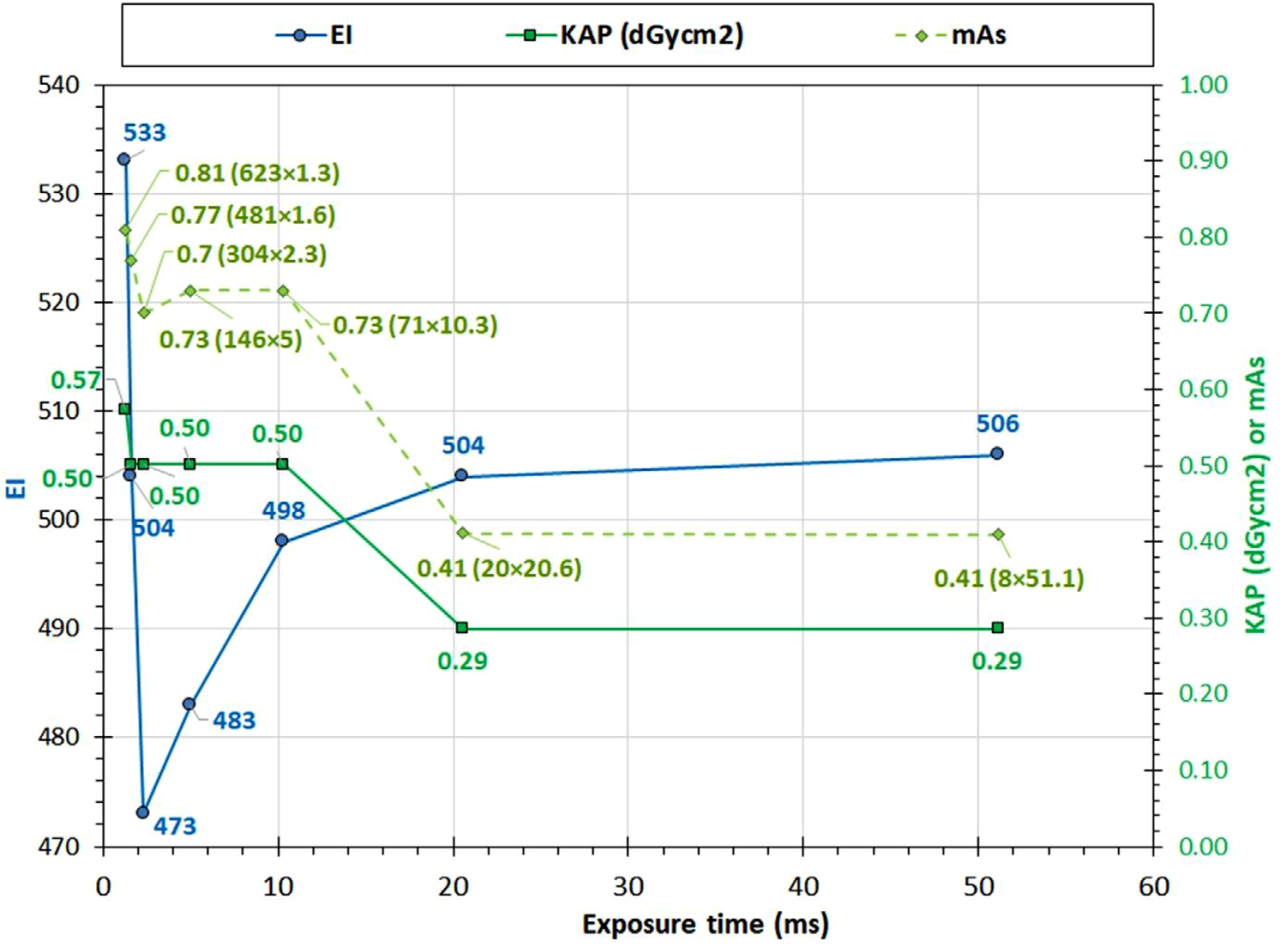
Philips system: EI versus exposure time (Chest AP examination). The data labels indicate the exact values of EI (left *Y*‐axis), KAP, and mAs (right *Y*‐axis). The labels for the mAs curve data points display the mAs value and, inside parentheses, the product of mA and exposure time in ms. EI, exposure index.

On the contrary, for the Philips system, it was seen that the effect of exposure time on EI was considerable. By increasing the selected mA value from 10 to 620 mA, the AEC system selected exposure times ranging from 51 down to 1.3 ms, presenting a peculiar variation pattern regarding EI but also mAs and KAP, that is shown in Figure 6. In this case, while EI differences were up to 14%, the differences in mAs and KAP were up to 100% (max/min ratio = 2). The mAs and KAP increased with shorter exposure times and as explained before, this can be attributed to the combined effect of two factors: the generator rise time and the AEC minimum response time.

#### 3.2.4 Effects of examination protocol and patient size selection

The preset Chest AP and Ribs AP examination protocols’ settings and the resulting exposure parameters and dose metrics are tabulated in Tables 1a and 1b. The system GE follows a rationale of adapting the examination setting to the different patient sizes (variable kVp, grid yes or no, sensor position variation, etc.). On the contrary, for the Philips system, the situation was much different, since the preset kVp of 125, the additional filter preset of 0.1 mm Cu + 1 mm A, and grid was used for all patient sizes. Except for newborns, where a constant mAs value was used, for the rest four patient sizes, the AEC was always activated (LR sensor selection), and the only difference is the dose level correction, being 0 for small patients, 1 for normal, and 2 for large and extra‐large patients.

**FIGURE 7:**
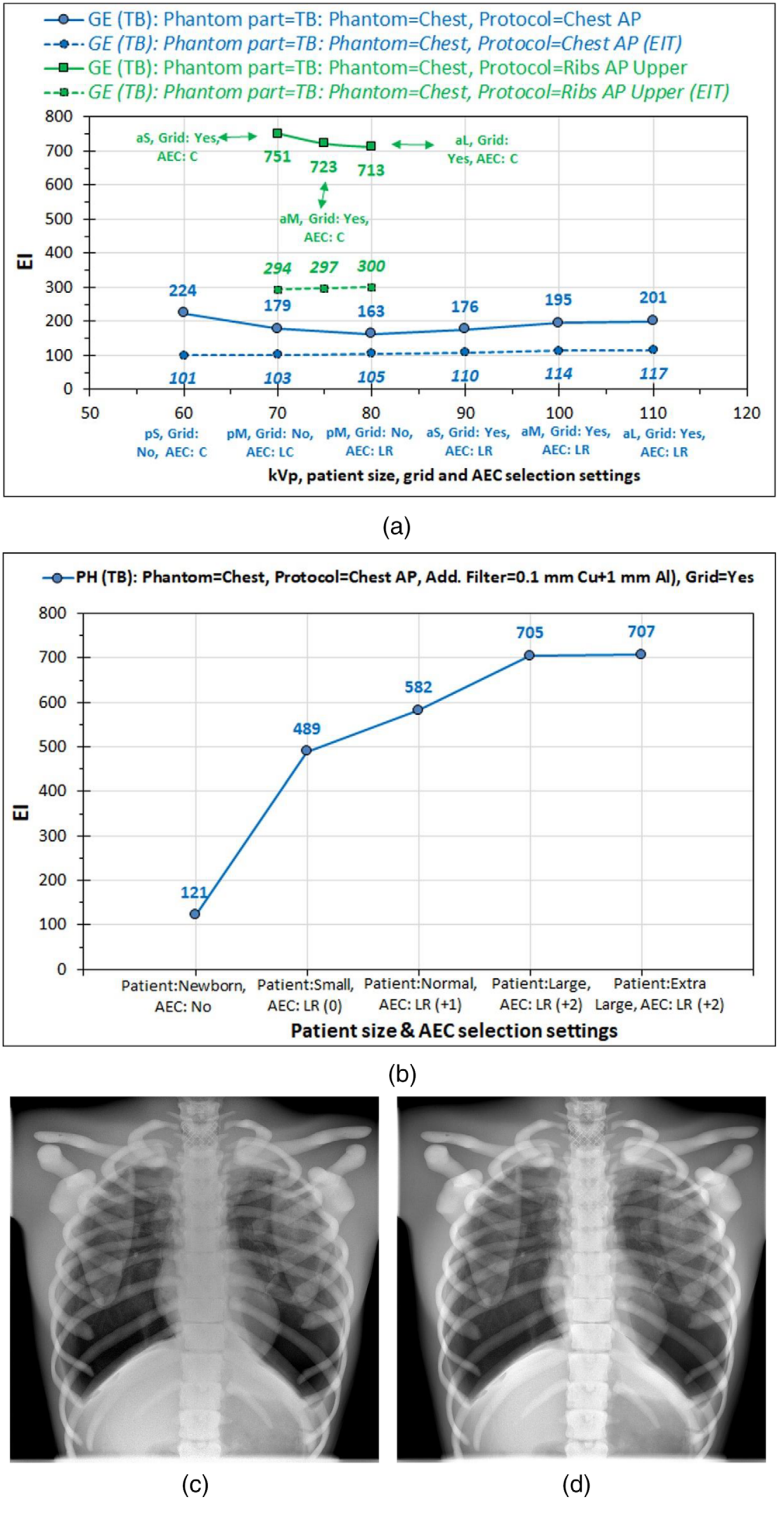
The effect of kVp, patient size, grid, and AEC selection settings on EI values and on image appearance. (a) GE (TB): EI and EI_T_ (dashed lines and labels in italics). (b) Philips (TB). (c) E system: Chest AP image appearance without grid (pL). (d) GE system: Chest AP image appearance with grid (aS). AEC, automatic exposure control; DI, deviation index; EI, exposure index; EI_T_, target exposure index.

For the GE system, as seen in Figure 7a, for larger patient sizes where the preset kVp is larger, there was a slight increase of EI (except for the first 2 data points) and EI_T_ values; however, EI and EI_T_ values are similar. This is despite what was expected for the first three data points of the blue‐colored curve, where no grid was used. While the removal of the grid affected much of the image appearance because of scatter radiation (see Figure 7c,d), EI doesn't seem to be reduced. However, if we compare these values with those of the green‐colored curve, where the same anatomy is imaged using only the central AEC sensor, which are much higher, it can be understood that this may be due to the fact that the central AEC sensor was activated alone or in combination with the left AEC sensor, forcing the EI to be increased due to the interference of the spinal cord. Thus, the interference of the central sensor explains why, for the first two data points of the blue curve (pS and pM), EI values are quite large despite that kVp is lower and the grid is off, while the lowest EI is observed for the third data point (pM). For the Philips system where images were acquired with the same kVp, filtration, and with the grid in place, the EI values increased with patient size because of the use of larger dose correction level. For newborns, the EI was very low as expected because the anthropomorphic phantom size is much larger than a newborn, and therefore the preset manual mAs are too low.

## 4 DISCUSSION

The results of the previous experiments with both the Al attenuator and the anthropomorphic phantom have unveiled the dependence of EI values, and therefore of the respective EI_T_ that should be set, on many parameters. While some of these have already been discussed in the literature,^6^, ^7^, ^8^, ^9^, ^10^, ^11^ others, like the dependence of EI on the exposure time shown in Figures 2a–c, 4i, and 6, according to our knowledge, have not been systematically described.

In clinical practice, many digital radiography systems operating under AEC, which employ the falling load technique, select the largest mA value allowed by the generator in order to minimize exposure time and thus minimize any unsharpness effects that could arise because of patient or patient organ involuntary moves (e.g., heartbeat). Given that modern digital radiography system generators have a power of 50 kW or larger, the AEC selected values can be 500 mA or even larger. In the case that a high kVp is used and the radiographed anatomy is not thick or dense enough, such high mA values will inevitably result in very small exposure times especially in systems where the IAK_T_ is adjusted at 2.5 µGy or lower. If the exposure times become comparable to the generator rise time and the minimum AEC response time, then the EI values will inadvertently be larger than expected. While this problem has been identified for TB, it can also appear in CB examinations when SID values similar to those in TB are used.

The only solution to this problem is to take all the necessary precautions so that exposure times that arise under AEC operation to be always larger than 10 ms, and the way to do this is to restrict the mA values in examination protocols where high kVp and/or short SID and/or no grid are used and/or thin anatomic regions are radiographed. This mA restriction can be normally incorporated in the preset examination protocol settings. This restriction also applies to the QC of AEC systems, since the EI is used as an indication of the correct AEC adjustment for different kVp and attenuator thicknesses. In these QC tests, care must be taken so that the kVp value selection is appropriate for the attenuator thickness used, or restrict the mA used, to avoid ending up with very short exposure times and overestimated EI values, which may be mistakenly considered as AEC malfunction or AEC maladjustment.

Another unexpected result was the large dependence of EI values on examination protocol post‐processing algorithm observed for the Philips system in Figure 5b. The fact that two ostensibly identical examination protocols, that is, the Ribs AP (1–7) and Ribs AP (8—12), gave greatly different EI values though all the rest of the acquisition parameters were the same, may suggest that EI values are not calculated by the raw image or that the clinical content detection is affected by the examination protocol. The much higher EI values for the Chest AP protocol compared to other protocols for the Philips system have also been observed in another phantom study.^11^ In any case, this observation explains why EI_T_ values for certain manufacturers should consider the post‐processing algorithm as well, even if the anatomy imaged appears to be the same. Thus, any changes in the post‐processing algorithm incorporated in an examination protocol may require a change in the default EI_T_ value as well.

Regarding the main issue, which is the use of EI as a measure of image quality by means of DI, the following remarks should be made. The first step is to determine proper EI_T_ values for each specific examination protocol, for example, after reviewing a large number of images to determine the median EI value and use it as EI_T_. Considering all the parameters related to the acquisition of clinical images described in this study and their possible variations from patient to patient (e.g., imaged anatomy, field size, examination post‐processing algorithm, kVp, AEC sensor combination, etc.), variations of up to ±50% should be considered highly probable. An EI value of 0.5, 0.625, 0.8, 1.25, 1.6, and 2 times the EI_T_ will result in DI values of −3, −2, −1, and 1, 2, and 3, respectively. Therefore, DI values within the range of −2 to +2 should be considered normal, as stricter limits would produce too many false red flags. Indeed, the specific GE radiographic system by default considers DI values within −3 to +2 variation range as normal (green indication), produces a warning (yellow indication) for DI values below −3 and down to −5 and above +2 and up to +4, and produces off‐limits warning (red indication) for DI values smaller than −5 and larger than +4.

## 5 CONCLUSION

Many parameters affect the EI calculation apart from IAK, with the manufacturer and imaged anatomy being among the main ones. Setting of the EI_T_ values should be done with caution on a per‐examination and manufacturer basis, since the values that apply for one digital system are not always applicable to another. Furthermore, when EI is used as an image quality tool in clinical images, a DI variation of at least ±2 should be allowed before a possibly meaningful red flag is activated.

## AUTHOR CONTRIBUTIONS

All authors substantially contributed to the conception or design of the research and interpreted results. Data acquisition and initial analysis were made by Ioannis A. Tsalafoutas and Shady AlKhazzam. Ioannis A. Tsalafoutas wrote the first draft, and the rest of the authors revised it for important intellectual content.

## CONFLICT OF INTEREST STATEMENT

The authors have no conflict of interest to state.
